# Severe Fever with Thrombocytopenia Syndrome Virus in Dogs, South Korea

**DOI:** 10.3201/eid2502.180859

**Published:** 2019-02

**Authors:** Jun-Gu Kang, Yoon-Kyoung Cho, Young-Sun Jo, Jeong-Byoung Chae, Young-Hoon Joo, Kyoung-Wan Park, Joon-Seok Chae

**Affiliations:** Seoul National University, Seoul, South Korea (J.-G. Kang, Y.-K. Cho, Y.-S. Jo, J.-B. Chae, J.-S. Chae);; Military Working Dog Training Center of the Republic of Korea Army, Chuncheon, South Korea (Y.-H. Joo, K.-W. Park)

**Keywords:** reverse transcription PCR, severe fever with thrombocytopenia syndrome virus, South Korea, dogs, viruses, phleboviruses, ticks, vector-borne infections

## Abstract

Of 103 serum samples collected from dogs in South Korea, 3 (2.9%) were positive for severe fever with thrombocytopenia syndrome virus (SFTSV) and 22 (21.4%) were positive for antibodies against SFTSV. A dog-derived isolate of SFTSV clustered with many South Korea SFTSV strains in the Japanese clade.

Severe fever with thrombocytopenia syndrome virus (SFTSV), a new tickborne phlebovirus of the *Phenuiviridae* family (previously *Bunyaviridae*), causes severe fever with thrombocytopenia syndrome (SFTS) in China, Japan, and the Republic of Korea (South Korea) (*1*). After identification of the first human case of SFTS in South Korea in 2013 (*1*), 335 cases (73 deaths; case-fatality rate 21.8%) were reported during 2013–2016 (*2*).

SFTSV is primarily transmitted through a tick bite. The *Haemaphysalis longicornis* tick is the main vector for SFTSV, promoting its circulation and transmission (*3*). Investigations have been conducted to determine the frequency of exposure of companion animals, wild animals, and livestock to SFTSV (*4**–**7*). Of particular importance, dogs are companion animals that have frequent contact with humans. Therefore, their infection status has major implications for public health. We isolated SFTSV from dog serum and determined the prevalence of SFTSV in dogs in South Korea.

We collected 103 serum samples during June–October 2016 from the following dog breeds: 42 Belgian Malinois, 58 German shepherds, and 3 Labrador retrievers. All dogs were military dogs in a training camp in Gangwon Province, South Korea, at the time of serum collection. The animals had no significant clinical signs associated with febrile disease. Information about body temperature, evidence of tick bites, blood chemistry, and complete blood count was unavailable.

Of the 103 samples, 3 (2.9%), obtained from dog 16, a German shepherd; dog 22, a Belgian Malinois; and dog 56, a German shepherd were positive for the small (S [346 bp]), medium (M [859 bp]), and L (large [1,165 bp]) segments of SFTSV by reverse transcription PCR (the L segment of dog 16 was not amplified). The sequences of the SFTSV S, M, and L segments differed from each other. The results of phylogenetic analysis of partial S, M, and L segments showed that sequences of SFTSV obtained from dogs were more related to strains from Japan than to strains from China (Appendix). Moreover, 22 (21.4%) samples were positive for SFTSV antibodies by immunofluorescence assay. SFTSV seroprevalence was 25.9% (15/58) for Belgian Malinois, 16.7% (7/42) for German shepherds, and 0% (0/3) for Labrador retrievers. Among seropositive dogs, 22.2% (12/54) were male and 20.4% (10/49) were female.

We used Vero cells to isolate the virus from positive serum. We observed cytopathic effect in only 1 of 3 positive samples. The results of phylogenetic analysis of the complete S segment indicated that the SFTSV strain isolated from dog 22 had not previously been isolated; this strain clustered with many SFTSV strains from South Korea and Japan (Figure).

**Figure:**
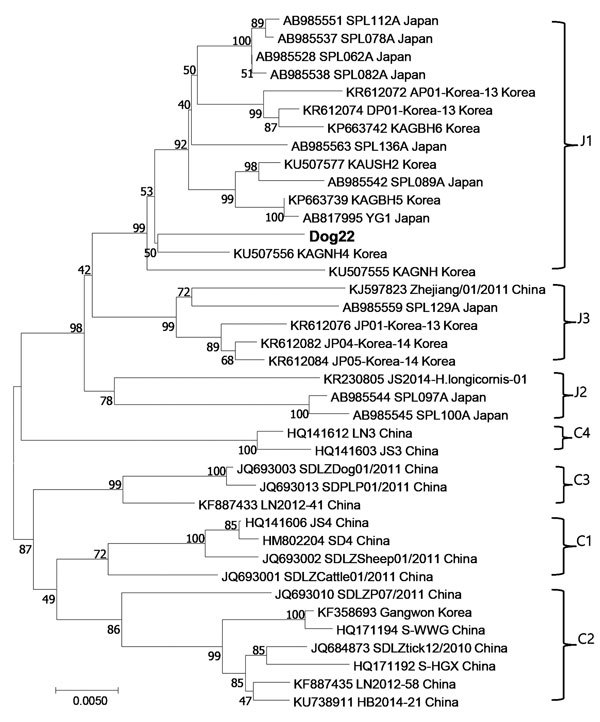
Phylogenetic analysis of severe fever with thrombocytopenia syndrome virus isolated from a military dog in South Korea (dog 22, bold) compared with reference viruses, based on the complete small segment. Evolutionary history was inferred using the maximum-likelihood method, based on the Kimura 2-parameter model (1,000 bootstrap replicates). The percentage of trees in which associated taxa clustered is shown next to the branches. The clades are designated by Japanese group. Scale bar indicates nucleotide substitutions per position.

*H*. *longicornis* ticks are the main vector of SFTSV and the dominant tick species collected from vegetation and animals in South Korea (*3*,*7*,*8*). However, because of the low SFTSV prevalence in ticks, mammalian hosts might be necessary for the circulation and maintenance of SFTSV in nature. Therefore, studies measuring the prevalence of SFTSV infection across various animal species have been undertaken (*4*–*7*). Only a few studies on SFTSV in dogs have been reported; these studies found that 1) the positive rates for SFTSV RNA were 5.3% (19/359) for domesticated dogs in China (*5*) and 0.2% (1/426) for shelter dogs in South Korea (*6*) and that 2) 28.9%–37.9% of domesticated dogs in China (*4*,*5*,*9*) and 13.9% of shelter dogs in South Korea (*10*) were seropositive for antibodies against SFTSV.

The detection rates of SFTSV RNA and antibodies in our study were 2.9% and 21.4%, respectively, which were higher than those observed in shelter dogs in South Korea (*6*). These results have 2 possible explanations. First, we collected samples during the summer, when dogs most easily and frequently have contact with ticks infected with SFTSV. In contrast, in the shelter dog study, the timing of sample collection was random and occurred throughout multiple seasons. Second, we drew serum from military dogs, which typically spend most of their time outside of the home; conversely, the shelter dog study examined small dogs that resided indoors before their relocation to a shelter.

Although we isolated only a few SFTSV strains from animals and our results could not represent all characteristics of SFTSV, our findings could indicate that SFTSV might not be host-specific and that various SFTSV clades circulate and are distributed in South Korea. Further studies continuously surveilling animals for SFTSV, along with whole-genome analysis of dog-derived Korean isolates of SFTSV, would help clarify the mechanisms of transmission and molecular evolution of SFTSV.

Appendix Additional information for severe fever with thrombocytopenia syndrome virus in dogs, South Korea.
