# Supplementary material for: Severe Fever with Thrombocytopenia Syndrome Virus in Dogs, South Korea
Source: Emerg Infect Dis. 2019 Feb;25(2):376–8. doi: 10.3201/eid2502.180859 (PMC6346443; doi:10.3201/eid2502.180859)
Supplement: Appendix — Additional information for severe fever with thrombocytopenia syndrome virus in dogs, South Korea. [file 18-0859-Techapp-s1.pdf]

Article DOI: https://doi.org/10.3201/eid2502.180859

Severe Fever with Thrombocytopenia Syndrome Virus in Dogs, Republic of Korea

Appendix

Methods

A total of 200 µL of serum was used for RNA extraction using the Gene-spin Viral DNA/RNA Extraction Kit (iNtRON Biotechnology, https://intronbio.com). Reverse transcription (RT), first PCR, and nested PCR were conducted to amplify S, M, and L segments of the SFTS viral RNA gene using a 1-step RT-PCR premix kit (Solgent, http://www.solgent.com/) with SFTSV genome–specific primer sets for PCR (Appendix Table). To identify the sequences for the SFTSV-positive samples, TA cloning with pGEM-T Easy Vectors (Promega, https://www.promega.com/) and sequencing using an automatic sequencer (3730xl capillary DNA Analyzer; Applied Biosystems, Foster City, CA, USA) were performed. The S segments (KY968712–KY9689714), M segments (KY968715–KY9689717), and L segments (KY968718 and KY9689719) of SFTSV were successfully sequenced and submitted to GenBank.

Vero cells were seeded in flat-bottomed 12-well plates at a concentration of 10^5 cells in 2 mL of Dulbecco's modified eagle medium (GE healthcare Life Sciences, https://www.gelifesciences.com/) containing 2% fetal bovine serum (Invitrogen, https://www.thermofisher.com/). After Vero cell monolayers were formed, 100 µL of positive serum was added into the 12-well plates. The plates were then incubated at 37°C with 5% CO_2 for 5–7 days. For identification of the isolated virus, viral RNA was extracted from the supernatants of passaged (passage 2–4) and infected cells. Sequencing of the complete S segment from isolated SFTSV was performed using the additional set of primers provided by Professor Lee (Jeju National University School of Medicine, Jeju, South Korea). Using the maximum-likelihood method in the MEGA 7 program (*2*) and based on the Kimura 2-parameter model, phylogenetic trees were constructed using the complete S segment sequences obtained from this study and GenBank.

An indirect immunofluorescence assay (IFA) was performed to detect antibodies against SFTSV. IFA slides were prepared using SFTSV-infected Vero cells. Vero cells were resuspended at 10^5 cell/well in media and were added to each well of 24 well slide glasses and incubated in 5% CO_2 for 16 h. Then the slides were fixed with 100% acetone for 10 min at –20°C. Then the diluted serum was added into IFA antigen slides and incubated in 5% CO_2 for 1 h. After washing with PBS, FITC-conjugated anti IgG (Sigma, https://www.sigmaaldrich.com/) was added to each well of the antigen slide and incubated in 5% CO_2 for 1 h. The visualization of the IFA slides was performed using EVOS® FL auto cell imaging system (Thermo Fisher Scientific, Inc., https://www.thermofisher.com/). The cutoff IFA value was determined based on the serial 2-fold dilution of positive and negative sample serum from 1:100 to 1:800 (data not shown). The goat serum that was positive against SFTSV (received from Korean Animal and Plant Quarantine Agency) was used as positive control.

References

1. Oh SS, Chae JB, Kang JG, Kim HC, Chong ST, Shin JH, et al. Detection of severe fever with thrombocytopenia syndrome virus from wild animals and Ixodidae ticks in the Republic of Korea. Vector Borne Zoonotic Dis. 2016;16:408–14. PubMed http://dx.doi.org/10.1089/vbz.2015.1848

2. Kumar S, Stecher G, Tamura K. MEGA7: Molecular Evolutionary Genetics Analysis version 7.0 for bigger datasets. Mol Biol Evol. 2016;33:1870–4. PubMed http://dx.doi.org/10.1093/molbev/msw054

Appendix Table. Nucleotide sequences of PCR primers and conditions for amplification of severe fever with thrombocytopenia syndrome virus genes

		Primer sequences, 5′–3′					
Target gene	PCR primers and conditions	Denaturation, °C/sec	Annealing, °C/sec	Extension, °C/sec	Cycle	PCR product size, bp	Reference
S segment	NP-2F	CATCATTGTCTTTGCCCTGA				461	(*1*)
	NP-2R	AGAAGACAGAGTTCACAGCA					
	Conditions	94/20	52/40	72/30	40		
	N2-F	AAYAAGATCGTCAAGGCATCA				346	
	N2-R	TAGTCTTGGTGAAGGCATCTT					
	Conditions	94/20	54/20	72/30	25		
M segment	MF1	CATCTGAAGCCAARTGYAGA				859	This study
	MR1	TTCATATTTCCGCTCCCTTG					
	Conditions	94/30	56/40	72/60	38		
L segment	LF1	GGCAGCAAAYCAGAAGAAAG				1,165	This study
	LR2	TTGTCTTCCATGTCGCTGAG					
	Conditions	94/30	56/40	72/60	38		

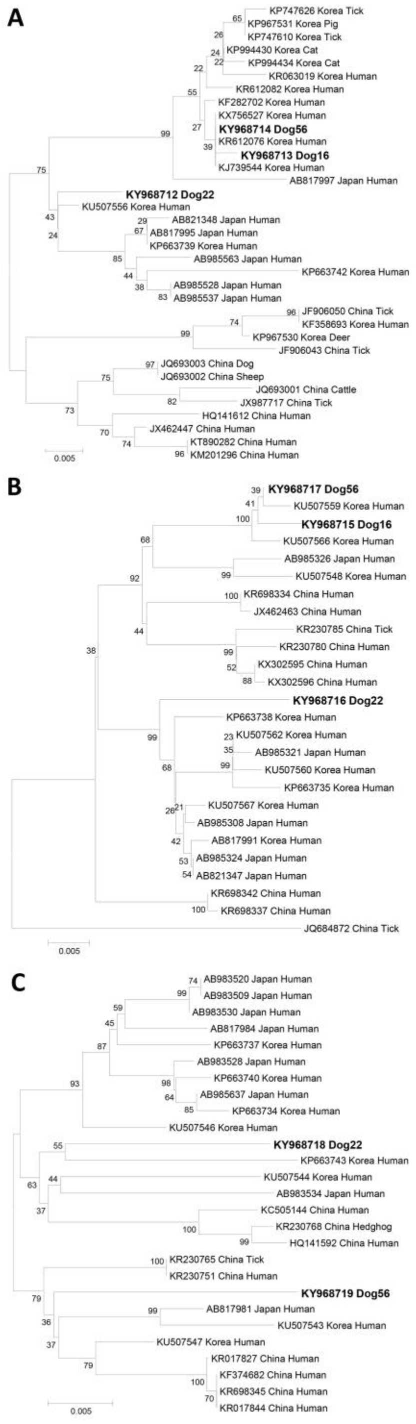

Appendix Figure 1. Phylogenetic analysis of severe fever with thrombocytopenia syndrome virus based on the partial small (A), medium (B), and large (C) segments. The sequences identified in this study are indicated by bold letters. Evolutionary history was inferred using the maximum-likelihood method, based on the Kimura 2-parameter model (1,000 bootstrap replicates). The percentage of trees with associated taxa clustered together is shown next to the branches. Scale bars indicates the number of nucleotide substitutions per position. The clades are designated by Japanese group.

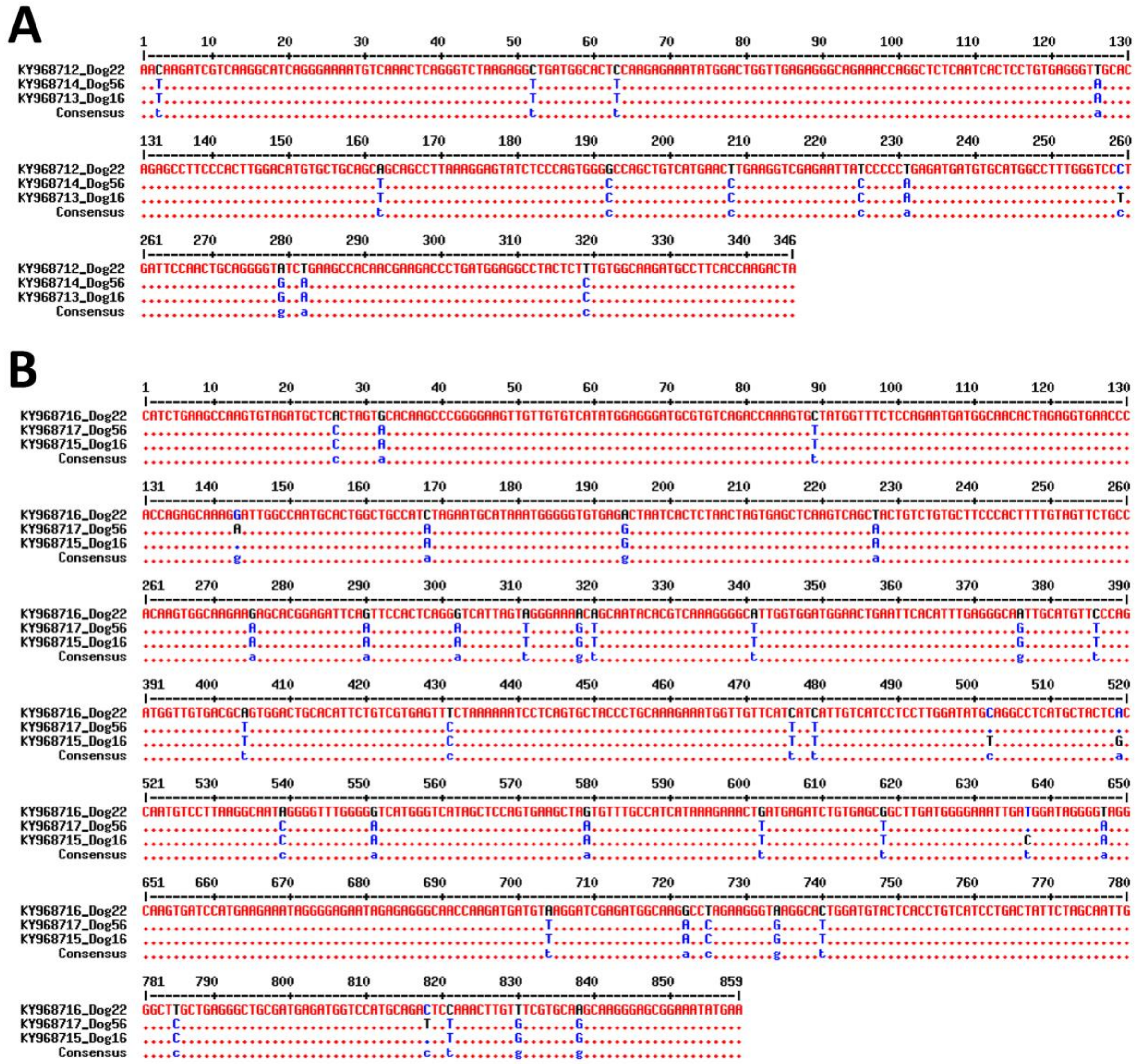

Appendix Figure 2. Genetic variation between 1 isolate (dog 22) and 2 amplified sequences (dog 16 and dog 56) on the partial small (A) and medium (B) segments.
